# Successful Treatment of Multilevel Tracheal Stenosis Post Blunt Chest Trauma in a Child by Early Bronchoscopic Balloon Dilatation: A Case Report

**DOI:** 10.3390/pediatric17060117

**Published:** 2025-11-04

**Authors:** Badar Al Dhouyani, Atqah AbdulWahab, Muna Maarafiya, Bilal Kabbara, Mutasim Abu-Hasan

**Affiliations:** Sidra Medicine, Ar-Rayyan, Doha P.O. Box 26999, Qatar; aabdulwahab@sidra.org (A.A.); mmohammed1@sidra.org (M.M.); bkabbara1@sidra.org (B.K.); mabuhasan@sidra.org (M.A.-H.)

**Keywords:** post blunt chest trauma, spirometry, fixed airway obstruction, biphasic stridor, tracheal stenosis, bronchoscopy

## Abstract

Background: Tracheal stenosis in children is a rare but potentially life-threatening condition. We report a case of multilevel tracheal stenosis in a child who sustained blunt chest trauma in a car accident. Case Presentation: The patient is an 11-year-old previously healthy boy who presented to the pediatric emergency room unconscious after being rolled over while seated unstrained inside a vehicle. A chest CT scan showed bilateral pulmonary contusions. He required intubation and mechanical ventilation initially but was noted to have biphasic stridor after extubation. He presented to the pediatric pulmonary clinic 2 weeks after discharge from the hospital with persistent stridor and shortness of breath on exertion. Spirometry revealed flattening of the inspiratory and expiratory limbs of the flow-volume loop, suggestive of fixed large airway obstruction. Direct laryngoscopy and bronchoscopy were performed and revealed multilevel tracheal stenosis. He was successfully treated with repeated bronchoscopic balloon dilatation with sustained improvement in symptoms and spirometry findings 8 months post final procedure. Conclusion: Tracheal stenosis should be suspected in children who sustain blunt chest trauma. Early recognition and treatment with bronchoscopic balloon dilatation can prevent long-term complications.

## 1. Introduction

Tracheal stenosis is a rare but serious condition in children, with an incidence ranging from 0.3% to 25% [1]. It can result from congenital airway defects or be acquired due to tracheal injury [2]. Congenital tracheal stenosis is extremely uncommon and often associated with other anomalies such as complete tracheal rings or vascular rings. Acquired tracheal stenosis is more frequently encountered in pediatric practice [3]. Common causes of acquired stenosis include prolonged intubation, which can cause mechanical and/or ischemic mucosal injury and subsequent scarring of the tracheal lumen [4]. Blunt trauma applied to the chest is another rare cause of tracheal injury.

Early recognition and management of tracheal injury are essential in preventing development of tracheal stenosis. Once stenosis occurs, prompt intervention is required to relieve symptoms and prevent progression. The treatment approach depends on the location, length, and severity of stenosis. Simple cases of tracheal stenosis caused by granulation tissue growth may respond well to laser ablation or balloon dilatation, while more severe cases caused by fibrotic scarring may require tracheal stenting, tracheal resection and re-anastomosis [5,6].

We report a successful outcome of early treatment of a multilevel tracheal stenosis using bronchoscopic balloon dilatation in a previously healthy child who sustained blunt chest trauma in a motor vehicle accident.

## 2. Case Presentation

The patient is an 11-year-old previously healthy male who presented with persistent noisy breathing and shortness of breath on exertion after recovering from a motor vehicle accident involving the rollover of a vehicle while the patient was seated unrestrained inside it. He was transferred to the pediatric emergency department unconscious, intubated, and mechanically ventilated. A chest CT revealed bilateral pulmonary contusion with no evidence of rib fractures or air leak (e.g., pneumomediastinum or pneumothorax). Head CT showed diffuse cerebral swelling with no skull fractures or intracranial bleed.

The patient was diagnosed with blunt chest and head trauma. He was admitted to the pediatric intensive care unit. His hospitalization lasted a total of 13 days, including 6 days on mechanical ventilation followed by 5 days on BiPAP and 2 days on O_2_ via a nasal cannula before he was finally weaned to room air.

**Figure 1:**
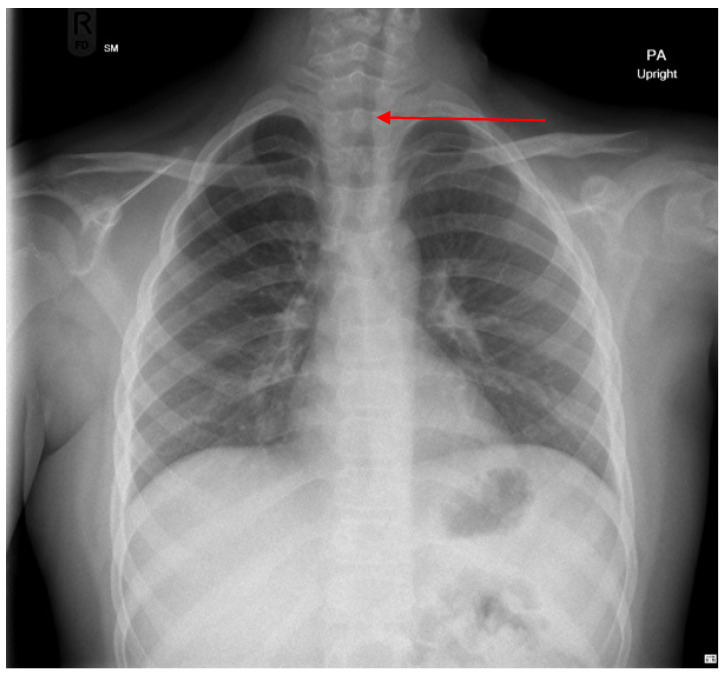
Chest X ray after extubation showing mid tracheal narrowing (red arrow).

The patient was noted to have biphasic stridor immediately after extubation. Upper airway evaluation showed normal vocal cord mobility and normal supraglottic structures. Chest X-ray revealed resolution of pulmonary infiltrates but showed mid-tracheal narrowing (Figure 1). He was discharged home after becoming clinically stable, to be followed as an outpatient.

**Figure 2:**
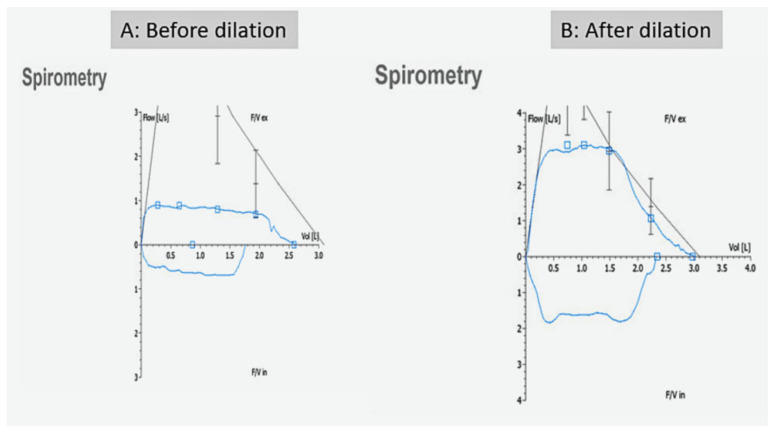
Spirometry before and after tracheal dilatation. (**A**): Flattening of the expiratory and inspiratory portions of the flow-volume loop which indicate fixed large airway obstruction. (**B**): flow-volume loop post treatment shows improved expiratory and inspiratory flows.

Two weeks after discharge, he presented to our pediatric pulmonary clinic with progressively worsening stridor associated with exertional dyspnea. Vital signs were normal, including normal O_2_ saturation on room air. Physical examination revealed biphasic stridor but with no signs of respiratory distress. Spirometry showed flattening of the inspiratory and expiratory limbs of the flow-volume loop indicative of fixed large airway obstruction (Figure 2).

Based on the above findings, rigid laryngoscopy and bronchoscopy were performed under general anesthesia, while the patient was kept spontaneously breathing. Suspension laryngoscopy was first introduced, with oxygen supplementation through the side port and intermittent endotracheal intubation when the patient desaturated. A zero-degree 4 mm × 30 cm endoscope was advanced into the airway. A multilevel Grade III tracheal stenosis in the mid-trachea was identified, eight tracheal rings above the carina, with a length of 3 cm and about 80–85% luminal narrowing at its most severe segment.

Balloon dilatation was performed as follows: a balloon catheter (TRACOE Aeris^®^, Ankara, Turkey) with a diameter of 10 mm and length of 30 mm was repeatedly inflated (four cycles) at a maximum pressure of 17ATM for a maximum of 2 min each cycle or until the patient developed O_2_ desaturation. Triamcinolone was injected into the tracheal wall to reduce scarring and restenosis.

**Figure 3:**
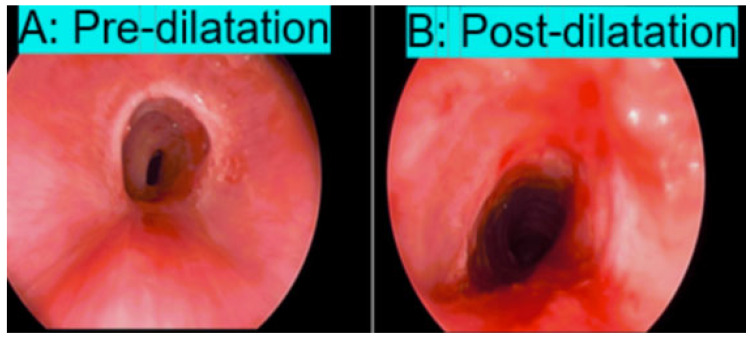
(**A**) Rigid bronchoscopy showing multilevel trachea stenosis due to circumferential scar tissue. (**B**) Bronchoscopy post balloon dilatation showing significant improvement in the tracheal stenosis.

The patient tolerated the procedure well and was transferred to a hospital bed not intubated and spontaneously breathing. The procedure resulted in significant improvement in tracheal lumen narrowing (Figure 3). Immediate symptomatic improvements in stridor and shortness of breath were noted immediately after the procedure. Repeat spirometry 2 weeks later showed significant improvement in the flow-volume loop findings (Figure 2).

Eight months post balloon dilatation, the patient developed stridor again but it was mild and with no dyspnea. A second bronchoscopic balloon dilatation was performed for a mild restenosis with an estimated degree of obstruction of 30–40%. The stridor was resolved immediately after the second balloon dilatation. The patient remained asymptomatic and had normal spirometry during a follow-up visit 8 months later. He was scheduled for yearly follow up in our pediatric pulmonary clinic, or more frequently if he were to become symptomatic.

Informed parental consent for this publication was obtained from the patient’s parent in accordance with the Sidra Medicine IRB policy.

## 3. Discussion

Acquired tracheal stenosis due to tracheal trauma is more common in children than tracheal stenosis due to congenital airway anomalies. Tracheal structure is normally well-protected by surrounding organs and tissues, which makes injuries uncommon. The incidence of tracheal injury varies widely, ranging from 0.3% to 25% [1]. Tracheal injury can vary in severity. Severe tracheal rupture occurs in 15–17% of tracheal injuries and is associated with a high mortality rate [2]. Close to 30% of affected patients die at the scene, and 60% of patients present later with tracheal stenosis [3].

Tracheal injury can occur from direct trauma, intubation, or blunt chest trauma. Direct tracheal trauma at the neck level may occur during motor vehicle accidents or due to collisions with objects such as cables. Intubation-related injury, on the other hand, is the most common iatrogenic cause of tracheal stenosis. Traumatic and/or prolonged intubation, especially with a large cuffed tube in hemodynamically unstable patients, can result in mucosal ischemia and scar formation leading to tracheal stenosis [4].

The exact cause of tracheal stenosis in our patient could not be determined with certainty. Possible causes include blunt chest trauma during the car accident or direct tracheal injury from the intubation, which was performed using a 7 mm cuffed tube (slightly larger than the recommended 6–6.5 mm for his age). Our patient had no evidence of direct tracheal injury, such as a tracheal bleeding or air leak (i.e., pneumomediastinum). Unfortunately, bronchoscopy was not performed immediately after intubation or shortly after extubation to evaluate for tracheal injury.

Blunt chest trauma is another possible cause of stenosis in our patient. Blunt chest trauma can cause tracheal injury via different mechanisms. One mechanism involves a sudden increase in intrathoracic pressure from chest compression against a closed glottis, leading to tracheal rupture, especially if the pressure exceeds the trachea’s tensile strength [5]. Blunt trauma can also cause compression and distortion of the trachea between the sternum and vertebral column, altering chest and tracheal dimensions and causing shear forces that disrupt the integrity of tracheal tissues [6,7]. Additionally, sudden acceleration–deceleration motion can produce shear stress between fixed airway points—the cricoid and the carina—leading to disruption, particularly 2.5–3 cm above the carina where shear forces are highest [8].

Tracheal injuries are usually diagnosed late. Clinical signs of tracheal injury can be absent or very subtle, even in the case of complete transection of the trachea if the surrounding tissues maintain airway continuity resulting in minimal symptoms. However, delayed diagnosis and intervention in these cases may result in development of tracheal stenosis from tight fibrotic strictures, with long-term consequences.

Varying degrees of tracheal stenosis can develop in intubated patients. Stauffer et al. reported that 19% of intubated patients had significant narrowing (>10% reduction in diameter), usually at the tube’s cuff level or at the subglottic level [9]. Longer intubation duration is thought to increase the risk of tracheal stenosis. However, the association of stenosis with prolonged intubation is not well established. Whited et al. found that tracheal stenosis was present in 12% of patients who were intubated >11 days, in 5% of patients intubated for 6–10 days, and in 2% of patients intubated for <6 days [10]. On the other hand, Stauffer et al. did not confirm this relationship [9].

CT imaging is a useful non-invasive tool for diagnosing tracheal injury and tracheal stenosis early. However, bronchoscopy remains essential for localization of the injury and its severity grading. In our case, CT failed to detect tracheal injury or tracheal stenosis. The clinical symptoms and the spirometry findings were the red herrings that prompted early bronchoscopy and intervention [11]. Prompt diagnosis and early treatment of tracheal stenosis reportedly lead to better outcomes. Delayed recognition can increase mortality by 20–30%, and up to 70% in some case series [12,13].

Endoscopic interventions are the mainstay treatment of early tracheal stenosis. Treatment of granulation tissue and/or scar tissue using balloon dilatation, lasers, electrocautery, and cryotherapy is commonly performed. Stenting of stenotic areas is reserved for severe and unresponsive forms of stenosis. Endoscopic balloon dilatation in many cases of tracheal stenosis in children has been associated with good short-term and long-term outcomes and improved quality of life [14,15]. Our patient responded well to balloon dilatation alone. A mild restenosis developed eight months later but was successfully treated with repeat balloon dilatation. The patient was evaluated again 8 months after the second balloon dilatations and had no signs or symptoms of restenosis.

Surgical tracheal resection and end-to-end anastomosis may be required in severe, complex or recurrent cases of tracheal stenosis [16]. Some studies suggest better long-term outcomes in these cases when compared to dilatation alone [17]. However, complications such as restenosis or anastomotic dehiscence have been reported in 5–6% of cases, typically within 3–22 weeks post surgery [1]. Tracheostomy can also help in stabilizing the airways during acute tracheal injury and as a therapeutic measure in certain severe and complex cases, or if other interventional measures are not feasible or have failed [4].

## 4. Conclusions

Tracheal stenosis can develop in children following blunt chest trauma and can present with chronic respiratory symptoms. Early recognition is critical for achieving favorable outcomes. Clinical signs, chest CT scan, spirometry, and bronchoscopy are helpful in early recognition. Endoscopic balloon dilatation is effective in treating the stenosis and can prevent long-term morbidity. In complex cases, surgical intervention or tracheostomy may be necessary.

## Data Availability

Any added data related to this case are available upon request from the corresponding author.
